# Genomic Insights into Gestational Weight Gain: Uncovering Tissue-Specific Mechanisms and Pathways

**DOI:** 10.21203/rs.3.rs-4427250/v1

**Published:** 2024-05-30

**Authors:** Elizabeth Jasper, Jacklyn Hellwege, Catherine Greene, Todd L Edwards, Digna Velez Edwards

**Affiliations:** Vanderbilt Unviersity Medical Center; Vanderbilt University Medical Center; Vanderbilt University; Division of Epidemiology, Department of Medicine, Vanderbilt Genetics Institute, Vanderbilt University Medical Center; Vanderbilt University Medical Center

**Keywords:** gestational weight gain, S-PrediXcan, gene expression, placenta

## Abstract

Increasing gestational weight gain (GWG) is linked to adverse outcomes in pregnant persons and their children. The Early Growth Genetics (EGG) Consortium identified previously genetic variants that could contribute to early, late, and total GWG from fetal and maternal genomes. However, the biologic mechanisms and tissue-Specificity of these variants in GWG is unknown. We evaluated the association between genetically predicted gene expression in five relevant maternal (subcutaneous and visceral adipose, breast, uterus, and whole blood) from GTEx (v7) and fetal (placenta) tissues and early, late, and total GWG using S-PrediXcan. We tested enrichment of pre-defined biological pathways for nominally (*P* < 0.05) significant associations using the GENE2FUNC module from Functional Mapping and Annotation of Genome-Wide Association Studies. After multiple testing correction, we did not find significant associations between maternal and fetal gene expression and early, late, or total GWG. There was significant enrichment of several biological pathways, including metabolic processes, secretion, and intracellular transport, among nominally significant genes from the maternal analyses (false discovery rate *p*-values: 0.016 to 9.37×10). Enriched biological pathways varied across pregnancy. Though additional research is necessary, these results indicate that diverse biological pathways are likely to impact GWG, with their influence varying by tissue and weeks of gestation.

## INTRODUCTION

Deviation from recommendations for gestational weight gain (GWG) has profound multigenerational consequences on pregnant persons and their children^1–4^. Roughly 70% of pregnant individuals in the United States (U.S.) do not fall within the recommended weight range^5,6^. Despite the significant public health burden of GWG, interventions, which largely focus on diet and physical activity during pregnancy, have had limited success in improving perinatal outcomes and are often difficult to adhere to outside of clinical trials^7^.

GWG is a complex phenotype, likely determined by a combination of genetic, metabolic, and environmental factors^6,8^. Weight gain, and its underlying biology, varies over the course of pregnancy. The modest weight gain in the first trimester is largely due to placental development and expansion of maternal blood volume, while larger weight gain in later trimesters is due to increasing fetal growth and development^9,10^. Twin studies have shown this weight gain is due, in part, to genetic variation with heritability ranging between 36% and 51% in studies conducted in nulliparous individuals^11^. Previous research has further investigated the genetic basis of gestational weight gain. A genome-wide association study (GWAS) investigated the genetic contribution to early, late, and total GWG using genetic variants in both fetal and maternal^12^. A single variant in offspring genomes reached genome-wide significance for total GWG but did not replicate. However, several variants that have been previously associated with related phenotypes, including increased body mass index (BMI), fasting glucose, and type 2 diabetes, were nominally significant. However, the biologic mechanisms and tissue-Specificity underlying the role of these variants in gestational weight gain remains unknown.

Our objective was to evaluate the association between tissue-Specific genetically predicted gene expression (GPGE) and GWG and interrogate biological pathways represented by the identified genes. We investigated associations in five tissues relevant to GWG using genotypes from pregnant persons. As the placenta is a key component of weight gain during pregnancy, we also examined the connection between gestational weight gain and gene expression in the placenta using fetal genotypes. Finally, since tissues that contribute to gestational weight gain are known to vary over the course of pregnancy, we evaluated these associations based on early, late, and total GWG.

## RESULTS

**Figure 1:**
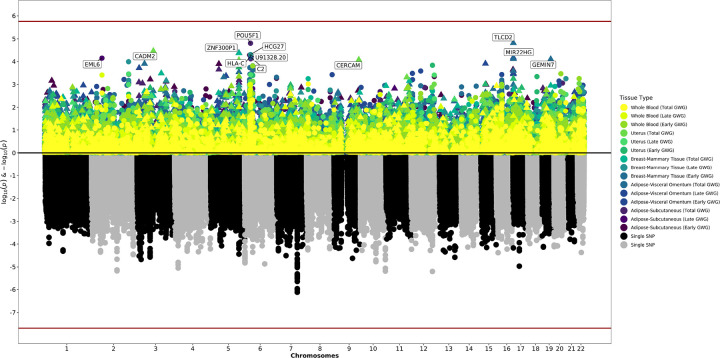
Miami Plot for Analyses Using Maternal Genotypes The bottom of the graphic is a Manhattan plot which displays significant SNPs from GWAS. The top of the graphic is the results from S-PrediXcan, with symbols now representing entire genes and their genetically determined expression levels. The x-axis are chromosomes. The y-axis is log and negative log p-values from the GWAS and S-PrediXcan analyses. Colors correspond to Specific tissues and definitions of gestational weight gain.

**Figure 2:**
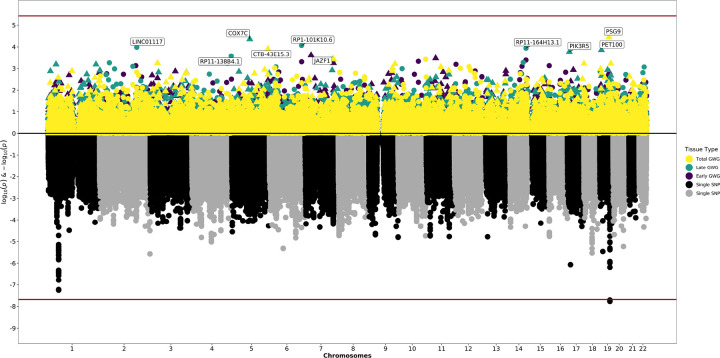
Miami Plot for Analyses Using Fetal Genotypes The bottom of the graphic is a Manhattan plot which displays significant SNPs from GWAS. The top of the graphic is the results from S-PrediXcan, with symbols now representing entire genes and their genetically determined expression levels. The x-axis are chromosomes. The y-axis is log and negative log p-values from the GWAS and S-PrediXcan analyses. Colors correspond to Specific tissues and definitions of gestational weight gain.

We tested the association of genetically predicted maternal or fetal gene expression with early, late, or total GWG in five maternal tissues and fetal placenta. We evaluated a total of 28,767 maternal gene-tissue pairs across subcutaneous and visceral adipose, breast, uterus, and whole blood; and a total of 14,145 fetal gene-tissue pairs in placenta (Supplemental Tables 1–2). There were no associations between genetically predicted gene expression in any tissue and early, late, or total GWG following multiple testing correction. There were, however, a number of nominally significant (*p*-value < 0.05) findings in maternal tissues and fetal placenta (Table 1, Figs. 1–2).

Genetically predicted gene expression in maternal tissues reveals important developmental and immune factors in early GWG, while results for late GWG reflect changes affecting cellular structures and interactions. *POU5F1* (β = −0.15, *p*-value = 1.55×10^− 5^), which plays a key role in embryonic development and stem cell pluripotency, had the most significant association with early GWG in maternal whole blood but was also nominally associated with late and total GWG in maternal whole blood. These results suggest that increased expression of *POU5F1* may be associated with decreased early GWG.

Genetically-predicted expression of multiple genes (*HCG27* [β = −0.15, *p*-value = 4.86×10^− 5^], *HLA-C* [β = 0.17, *p*-value = 5.87×10^− 5^], *C2* [β = 0.24, *p*-value = 7.68×10^− 5^]) involved in the immune system was also most strongly tied to early GWG in maternal tissues. Among early, late, and total GWG, *HLA-C* was nominally significant for all maternal tissues except subcutaneous adipose, while *C2* was nominally significant for only whole blood. *HCG27* was more consistently tied to predicted expression changes in maternal adipose tissue, with nominal associations detected for early, late, and total GWG. The leading maternal associations with late GWG were genetically-predicted expression of genes involved in cell aggregation, adhesion, and proliferation, with additional roles in splicing and membrane rigidity (*CADM2* [β = −0.29, *p*-value = 3.39×10^− 5^], *ZNF300P1* [β = 0.08, *p*-value = 4.11×10^− 5^], *TLCD2* [β = −0.26, *p*-value = 7.73×10^− 5^], *GEMIN7* [β = 0.93, *p*-value = 7.98×10^− 5^]).

In fetal placenta, genetically-predicted expression of genes involved in transcription and cell proliferation (*JAZF1* [β = 0.08, *p*-value = 2.44×10^− 4^], *ALX4* [β = −0.16, *p*-value = 3.35×10^− 4^], *TLC1A* [β = −0.13, *p*-value = 4.10×10^− 4^]) were linked to early GWG, while expression of genes with mitochondria functions or previously implicated in cancers (*COX7C* [β = 0.32, *p*-value = 4.34×10^− 5^], *PET100* [β = 0.14, *p*-value = 1.38×10^− 4^], *LINC01117* [β = 0.19, *p*-value = 1.03×10^− 4^], *RP11–164H13.1* [β = 0.18, *p*-value = 1.14×10^− 4^]) were tied to late GWG. Fetal placenta expression of *PSG9* (β = −0.11, *p*-value = 3.57×10^− 5^) had the most significant association with total GWG.

We performed gene set enrichment analyses incorporating nominally significant genes from our maternal and fetal analyses using FUMA’s gene2func tool. In maternal tissues, intracellular, vesicle-mediated, intracellular protein transport, Golgi vesicle transport, and establish of protein localization processes were associated with early GWG (Supplemental Fig. 1). Purine nucleotide binding was an enriched function for both early and late GWG. Sulfur compound metabolic processes were associated with total GWG (Supplemental Fig. 2). Nominally significant genes from the maternal early and total GWG analysis have also been previously associated with several related phenotypes, including waist and hip circumferences adjusted for body mass index (Supplemental Fig. 3). There was no enrichment of processes or functions in nominally significant genes from fetal analyses.

## DISCUSSION

We tested the association between GWG and tissue Specific GPGE using five relevant maternal tissues and the fetal placenta. Many nominally significant associations demonstrated strong biologic plausibility. In maternal analyses, genes involved in immune system were among the top associations for early GWG; while top associations for late GWG included genes with function in cell aggregation, adhesion, and proliferation. There was also significant enrichment of several biologic pathways, such as metabolic processes, secretion, and intracellular transport, among nominally significant genes from the maternal analyses. The enriched biological pathways varied by pregnancy stage (e.g., early vs late GWG). In fetal analyses, placental expression of a gene encoding a pregnancy-Specific protein was the leading association with total GWG. In addition to the top genes’ involvement in relevant biological processes, several of these genes have been previously tied to relevant phenotypes, including BMI and waist to hip ratio. After correcting for multiple testing, however, we did not find statistically significant associations between maternal and fetal tissue-Specific gene expression and early, late, or total of GWG.

Previous research has found a moderate genetic contribution to weight gain. The only GWAS for GWG to date, from which summary statistics for our study were obtained, concluded that roughly 20% of the variability in GWG can be explained by common maternal variants, with variants from the fetal genome contributing a much smaller amount^12^. Other genetic studies, investigating only candidate variants or genes have also been performed. Previous research has found associations with variants tied to obesity and diabetes, including SNPs in *KCNQ1*, *PPARG*, and *GNB3*^12–19^. A recent study found risk variants in *TMEM18* and *GNB3* are more frequent in females with increased GWG, a variant in *GNPDA2* was more frequent in those with adequate GWG, and a variant in *LEPR* was more common in individuals with decreased GWG^17^. Unfortunately, there are currently no gene expression prediction models for *KCNQ1* and *PPARG* in the maternal tissues. *GNB3, TMEM18, GNPDA2*, and *LEPR* was not significant in any tissue in the maternal analyses. *KCNQ1, PPARG, GNB3, GNPDA2*, and *LEPR* were also not significant in any fetal analyses. *TMEM18* was nominally significant in only the early GWG fetal analysis (β = 0.04, *p*-value = 0.03).

The Warrington et al. study found a single variant in offspring genome that was significantly associated with total gestational weight gain: *PSG5*^12^. Interestingly, the top nominally significant association in the fetal placenta analysis for total gestational weight gain was another pregnancy-Specific beta-1 glycoprotein: *PSG9. PSGs* are produced by placental trophoblasts and secreted into the maternal bloodstream in increasing amounts throughout pregnancy^20^. The function of these proteins is not fully known, but they have several hypothesized roles, including modulation of the maternal immune system to avoid rejection of the fetus. Previous studies reveal an association between these proteins and preeclampsia and fetal growth restriction^21,22^. Our findings are consistent with previous work on PSGs and fetal growth restriction indicating that increased expression of *PSG9* is associated with decreased total GWG.

Overexpression of *JAZF1*, the top nominally significant association with early GWG in the placenta, has been shown to reduce body weight gain and regulate lipid metabolism in mice models^23^. In contrast, our results demonstrate increased placental expression was associated with increased GWG in early pregnancy. *JAZF1* has been previously implicated in GWAS for type 2 diabetes, body fat distribution, and body mass index^24–27^. The top suggestive associations in early (*POU5F1*-whole blood), late (*CADM1*-breast), and total (*TLCD2*-subcutaneous adipose) GWG using maternal genotypes have also been tied to related traits, including BMI, BMI-adjusted hip circumference, type 2 diabetes, and waist to hip ratio^26,28–32^.

We observed additional associations that had strong biological plausibility in our analyses stratified by time of the GWG measurement. For example, there were many maternal immune system genes (*HCG27, HLA-C, C2*, etc.) nominally associated with early GWG. There is a strong biological basis for this association, as the maternal immune system must adapt early in pregnancy to ensure the fetus is not rejected. In the GWAS Catalog, previous associations for these genes notably include obesity traits like waist to hip ratio, in addition to other immune and blood traits and autoimmune disorders such as lupus and psoriasis. *POU5F1*, which plays a key role in embryonic development and stem cell pluripotency, had the most significant association with early GWG in maternal analyses. In contrast, expression of genes involved in cell aggregation, adhesion, and proliferation, as well as splicing and membrane rigidity (*CADM2, ZNF300P1, TLCD2, GEMIN7*) were the leading associations with late GWG in maternal analyses. In the fetal placenta, genetically predicted expression of genes involved in transcription and cell proliferation (*JAZF1, ALX4, TLC1A*) were linked to early GWG. Enriched biologic pathways and functions also differed based on period of GWG, though purine nucleotide binding was enriched in both early and late GWG of maternal analyses and fetal analyses did not display any enrichment in processes or functions.

There are several considerations related to the data utilized in this study. First, our threshold for statistical significance accounts for the number of gene-tissue pairs tested. This threshold could be overly stringent as it does not take into account the correlation structures between coordinated expression both within tissues and for the same genes across tissues. Significant results reported in this analysis could be overly conservative and not detect additional true effects that did not reach this threshold. GTEx and the corresponding tissue-Specific prediction models for maternal analyses utilize samples of non-diseased tissues from both female and male donors with a variety of races and ages. Investigation using sex-Specific models and/or reproductive females only could yield additional insight. Sample sizes varied based on tissue and ranged from 70 to over 400, which could also impact our power to detect associations. We utilized publicly available data from a GWAS of over 10,000 females and 7,000 offspring^12^. All study participants were of European ancestry. Future research should include more diverse populations. Additionally, GPGE models are only available for the fetal portion of the placenta. Creation of models using the maternal side of the placenta would be beneficial for direct comparison.

In conclusion, many of the genes whose expression was nominally significant possess functions that are likely relevant to weight gain in pregnancy. Associations varied by time of weight gain, broadly mirroring the events during pregnancy. For example, immune genes were top associations in early GWG, a time when maternal immune systems must quickly adapt to ensure the fetus is not rejected. Several of these genes have also been previously implicated in GWAS of related traits. These results further support that diverse biological pathways impact GWG, inferring that their influence is likely to vary based on individual and tissue, as well as over the course of pregnancy.

## MATERIALS AND METHODS

### Genome-Wide Association Study Data

GWAS summary statistics were obtained from a genome-wide meta-analysis on gestational weight gain. Data on gestational weight gain has been contributed by the Early Growth Genetics (EGG) Consortium and has been downloaded from www.egg-consortium.org^12^. The previous study used maternal and fetal genotypes to investigate three gestational weight gain definitions: early, late, and total. Early was calculated as the difference between pre-/early pregnancy weight and weight measured any time between 18 and 20 completed weeks divided by length of gestation in weeks at last measurement. Late gestational weight gain equaled the difference between 18- and 20-week measurement and last gestational weight measure at or after 28 weeks divided by the difference in gestational age in completed weeks between these two measurement times. Finally, total gestational weight gain was equivalent to the last gestational weight (at or after 28 weeks gestation) prior to delivery minus pre-/early-pregnancy weight divided by length of gestation in weeks at last measurement. Using maternal genotypes, the analyses contained up to 7,704, 7,681, or 10,555 individuals for early, late, and total gestational weight gain, respectively. For analyses using fetal genotypes, there were up to 8,552, 8,625, or 16,469 individuals in the meta-analyses of early, late, or total gestational weight. All singleton pregnancies that resulted in a term delivery where the child did not have a known congenital anomaly were included. All individuals in the 20 included pregnancy and birth cohorts were of European origin^12^.

### Placental Gene Expression Models

We have previously described our methods for creation of a placental gene expression model^33,34^. Briefly, placental gene expression and expression quantitative trait loci (eQTLs) were evaluated and computed using published gene expression data from the Rhode Island Child Health Study^35^. Gene expression data on 150 samples were derived from placenta tissue excluding maternal decidua and processed using whole transcriptome RNAseq. As previously described, whole genome genotyping (Illumina MEGAex Array, Illumina Inc., San Diego, CA) was used for generating eQTLs^35^. Genetically predicted gene expression models were created using eQTL summary statistics. Within each gene, total eQTLs were filtered by a false discovery rate (FDR)-adjusted p-value less than 0.1. Linkage disequilibrium clumping was performed (0.1 r^2^ and 250 kilobase window). To retain only genes with considerable genetic regulation in placenta, the variance explained by each eQTL single nucleotide polymorphism (SNP) was calculated and the sum of SNP variances was computed for each gene. Genes were then ordered by expression variance explained and those with variance of less than 0.01 or greater than two were excluded from the prediction models. Final models used 25,885 genetic variants associated with expression of 15,154 genes^33,34^. The placenta model was only used for analyses using fetal genotypes.

### S-PrediXcan Analyses

Gene expression was estimated in placental tissue using the gestational weight gain GWAS summary statistics and S-PrediXcan. This method tests for association between the outcome and tissue-Specific genetically determined component of expression for genes using SNP-level association and eQTL summary statistics^36^. For analyses, S-PrediXcan was also used to obtain gene expression estimates in five relevant maternal tissues (subcutaneous and visceral adipose, breast, uterus, and whole blood) using existing models from the Genotype Tissue Expression (GTEx v7) project (predictdb.org). We also evaluated associations between genetically predicted gene expression in the placenta and early, late, and total gestational weight gain using summary statistics from the GWAS using fetal genotypes. Bonferroni corrected *p*-values were used to determine significant gene associations significant (*p*-value < 1.74×10^− 6^ for all maternal analyses, *p*-value < 3.58×10^− 6^ for early and late gestational weight gain using fetal genotypes, and *p*-value < 3.54×10^− 6^ for total gestational weight gain using fetal genotypes), thought associations with *p*-values less than 0.05 were considered nominally significant. All reported effect sizes equate to increasing predicted gene expression. We defined genes as previously implicated in GWAS studies if they were reported and mapped in the GWAS Catalog for each outcome definition. All results are based on publicly available data and do not constitute human subjects research.

### Functional annotation

The annotation tool Functional Mapping and Annotation of Genome-Wide Association Studies (FUMA) gene2func was used to test for gene enrichment in cell types and pre-defined biological pathways for all significant and nominally associations^37,38^.

## Figures and Tables

**Table 1:** Top Associations for All Analyses

Genotype	GWG	Tissue	Gene	Effect (SD of GWG per SD of gene expression)	Standard Error	P-value
**Maternal**	**Early**	Whole blood	*POU5F1*	−0.15	0.04	1.55 × 10
		Adipose (visceral)	*HCG27*	−0.15	0.04	4.86 × 10
		Adipose (subcutaneous)	*U91328.20*	0.32	0.08	5.17 × 10
		Whole blood	*HLA-C*	0.17	0.04	5.87 × 10
		Whole blood	*C2*	0.24	0.06	7.68 × 10
	**Late**	Breast	*CADM2*	−0.29	0.07	3.39 × 10
		Adipose (subcutaneous)	*ZNF300P1*	0.08	0.02	4.11 × 10
		Adipose (visceral)	*ZNF300P1*	0.07	0.02	4.30 × 10
		Adipose (subcutaneous)	*TLCD2*	−0.26	0.06	7.74 × 10
		Adipose (subcutaneous)	*GEMIN7*	0.93	0.24	7.98 × 10
	**Total**	Adipose (subcutaneous)	*TLCD2*	−0.25	0.06	1.52 × 10
		Whole blood	*C2*	0.20	0.05	6.48 × 10
		Whole blood	*MIR22HG*	0.26	0.06	6.75 × 10
		Uterus	*EML6*	−0.11	0.03	7.16 × 10
		Whole blood	*TLCD2*	0.23	0.06	7.55 × 10
**Fetal**	**Early**	**Placenta**	*JAZF1*	0.08	0.02	2.44 × 10^4^
			*ALX4*	−0.16	0.05	3.35 × 10^4^
			*TCL1A*	−0.12	0.04	4.10 × 10^4^
			*C10orf129*	−0.17	0.05	4.68 × 10^4^
			*Rp11–475C16.1*	0.18	0.05	4.91 × 10^4^
	**Late**		*COX7C*	0.32	0.08	4.34 × 10
			*RP1–101K10.6*	−0.22	0.06	8.47 × 10
			*LINC01117*	0.19	0.05	1.03 × 10^4^
			*RP11–164H13.1*	0.18	0.05	1.14 × 10^4^
			*PET100*	0.14	0.04	1.38 × 10^4^
	**Total**		*PSG9*	−0.11	0.03	3.57 × 10
			*CTB-43E15.3*	0.06	0.01	1.22 × 10^4^
			*RNU6–1154P*	−0.13	0.04	3.64 × 10^4^
			*MGMT*	0.08	0.02	3.77 × 10^4^
			*AC018359.1*	0.10	0.03	5.69 × 10^4^

* GWG, gestational weight gain; SD, standard deviation

## Data Availability

Publicly available data analyzed during this study are included in published studies. Data on gestational weight gain has been contributed by the Early Growth Genetics (EGG) Consortium and has been downloaded from www.egg-consortium.org. Any additional information and data are available upon reasonable request. The data and materials can be shared by Dr. Elizabeth A. Jasper (elizabeth.jasper@vumc.org) upon reasonable request.

## References

[1] Goldstein R. F. Association of Gestational Weight Gain With Maternal and Infant Outcomes: A Systematic Review and Meta-analysis. JAMA 317, 2207–2225, doi:10.1001/jama.2017.3635 (2017). 28586887 PMC5815056

[2] LifeCycle Project-Maternal O. Association of Gestational Weight Gain With Adverse Maternal and Infant Outcomes. JAMA 321, 1702–1715, doi:10.1001/jama.2019.3820 (2019). 31063572 PMC6506886

[3] Mamun A. A., Mannan M. & Doi S. A. Gestational weight gain in relation to offspring obesity over the life course: a systematic review and bias-adjusted meta-analysis. Obes Rev 15, 338–347, doi:10.1111/obr.12132 (2014). 24321007

[4] Nehring I., Schmoll S., Beyerlein A., Hauner H. & von Kries R. Gestational weight gain and long-term postpartum weight retention: a meta-analysis. Am J Clin Nutr 94, 1225–1231, doi:10.3945/ajcn.111.015289 (2011). 21918221

[5] Deputy N. P., Sharma A. J. & Kim S. Y. Gestational Weight Gain - United States, 2012 and 2013. MMWR Morb Mortal Wkly Rep 64, 1215–1220, doi:10.15585/mmwr.mm6443a3 (2015). 26540367 PMC4862652

[6] IOM (Institute of Medicine) and NRC (National Research Council). in Weight Gain During Pregnancy: Reexamining the Guidelines The National Academies Collection: Reports funded by National Institutes of Health (eds Rasmussen K. M. & Yaktine A. L.) Ch. 3, (National Academies Press (US), 2009). 20669500

[7] Rogozinska E. Gestational weight gain outside the Institute of Medicine recommendations and adverse pregnancy outcomes: analysis using individual participant data from randomised trials. BMC Pregnancy Childbirth 19, 322, doi:10.1186/s12884-019-2472-7 (2019). 31477075 PMC6719382

[8] National Research Council. Influence of pregnancy weight on maternal and child health: workshop report. (Washington, DC, 2007).

[9] Gilmore L. A., Klempel-Donchenko M. & Redman L. M. Pregnancy as a window to future health: Excessive gestational weight gain and obesity. Semin Perinatol 39, 296–303, doi:10.1053/j.semperi.2015.05.009 (2015). 26096078 PMC4516569

[10] Hytten F. E. & Leitch I. The physiology of human pregnancy. 2nd edn, (Blackwell Scientific Publications, 1980).

[11] Andersson E. S. Heritability of gestational weight gain–a Swedish register-based twin study. Twin Res Hum Genet 18, 410–418, doi:10.1017/thg.2015.38 (2015). 26111621

[12] Warrington N. M. Maternal and fetal genetic contribution to gestational weight gain. Int J Obes (Lond) 42, 775–784, doi:10.1038/ijo.2017.248 (2018). 28990592 PMC5784805

[13] Dishy V. G-protein beta(3) subunit 825 C/T polymorphism is associated with weight gain during pregnancy. Pharmacogenetics 13, 241–242, doi:10.1097/00008571-200304000-00009 (2003). 12668921

[14] Groth S. W., LaLonde A., Wu T. & Fernandez I. D. Obesity candidate genes, gestational weight gain, and body weight changes in pregnant women. Nutrition 48, 61–66, doi:10.1016/j.nut.2017.11.008 (2018). 29469022 PMC5857243

[15] Marginean C. The relationship among GNB3 rs5443, PNPLA3 rs738409, GCKR rs780094 gene polymorphisms, type of maternal gestational weight gain and neonatal outcomes (STROBE-compliant article). Medicine (Baltimore) 98, e16414, doi:10.1097/MD.0000000000016414 (2019). 31305457 PMC6641780

[16] Stuebe A. M. Obesity and diabetes genetic variants associated with gestational weight gain. Am J Obstet Gynecol 203, 283 e281–217, doi:10.1016/j.ajog.2010.06.069 (2010). PMC3222335 20816152

[17] Mikolajczyk-Stecyna J. Genetic risk score for gestational weight gain. Eur J Obstet Gynecol Reprod Biol 294, 20–27, doi:10.1016/j.ejogrb.2023.12.031 (2024). 38184896

[18] Beysel S. Maternal genetic contribution to pre-pregnancy obesity, gestational weight gain, and gestational diabetes mellitus. Diabetol Metab Syndr 11, 37, doi:10.1186/s13098-019-0434-x (2019). 31114636 PMC6518700

[19] Groth S. W. & Morrison-Beedy D. GNB3 and FTO Polymorphisms and Pregnancy Weight Gain in Black Women. Biol Res Nurs 17, 405–412, doi:10.1177/1099800414561118 (2015). 25510251 PMC5527338

[20] Moore T. Pregnancy-Specific glycoproteins: evolution, expression, functions and disease associations. Reproduction 163, R11–R23, doi:10.1530/REP-21-0390 (2022). 35007205

[21] Tiensuu H. Risk of spontaneous preterm birth and fetal growth associates with fetal SLIT2. PLoS Genet 15, e1008107, doi:10.1371/journal.pgen.1008107 (2019). 31194736 PMC6563950

[22] Kandel M. PSG7 and 9 (Pregnancy-Specific beta-1 Glycoproteins 7 and 9): Novel Biomarkers for Preeclampsia. J Am Heart Assoc 11, e024536, doi:10.1161/JAHA.121.024536 (2022). 35322669 PMC9075453

[23] Jang W. Y. Overexpression of Jazf1 reduces body weight gain and regulates lipid metabolism in high fat diet. Biochem Biophys Res Commun 444, 296–301, doi:10.1016/j.bbrc.2013.12.094 (2014). 24380856

[24] Pulit S. L. Meta-analysis of genome-wide association studies for body fat distribution in 694 649 individuals of European ancestry. Hum Mol Genet 28, 166–174, doi:10.1093/hmg/ddy327 (2019). 30239722 PMC6298238

[25] Rask-Andersen M., Karlsson T., Ek W. E. & Johansson A. Genome-wide association study of body fat distribution identifies adiposity loci and sex-Specific genetic effects. Nat Commun 10, 339, doi:10.1038/s41467-018-08000-4 (2019). 30664634 PMC6341104

[26] Replication D. I. G. Genome-wide trans-ancestry meta-analysis provides insight into the genetic architecture of type 2 diabetes susceptibility. Nat Genet 46, 234–244, doi:10.1038/ng.2897 (2014). 24509480 PMC3969612

[27] Vujkovic M. Discovery of 318 new risk loci for type 2 diabetes and related vascular outcomes among 1.4 million participants in a multi-ancestry meta-analysis. Nat Genet 52, 680–691, doi:10.1038/s41588-020-0637-y (2020). 32541925 PMC7343592

[28] Kichaev G. Leveraging Polygenic Functional Enrichment to Improve GWAS Power. Am J Hum Genet 104, 65–75, doi:10.1016/j.ajhg.2018.11.008 (2019). 30595370 PMC6323418

[29] Justice A. E. Genome-wide meta-analysis of 241,258 adults accounting for smoking behaviour identifies novel loci for obesity traits. Nat Commun 8, 14977, doi:10.1038/ncomms14977 (2017). 28443625 PMC5414044

[30] Vogelezang S. Novel loci for childhood body mass index and shared heritability with adult cardiometabolic traits. PLoS Genet 16, e1008718, doi:10.1371/journal.pgen.1008718 (2020). 33045005 PMC7581004

[31] Sakaue S. A cross-population atlas of genetic associations for 220 human phenotypes. Nat Genet 53, 1415–1424, doi:10.1038/s41588-021-00931-x (2021). 34594039 PMC12208603

[32] Christakoudi S., Evangelou E., Riboli E. & Tsilidis K. K. GWAS of allometric body-shape indices in UK Biobank identifies loci suggesting associations with morphogenesis, organogenesis, adrenal cell renewal and cancer. Sci Rep 11, 10688, doi:10.1038/s41598-021-89176-6 (2021). 34021172 PMC8139988

[33] Hellwege J. N. Association of genetically-predicted placental gene expression with adult blood pressure traits. Circulation: Genomic and Precision Medicine (2023). 10.1097/HJH.0000000000003427 PMC10287061 37016918

[34] Jasper E. A. Genetically-predicted placental gene expression is associated with birthweight and adult body mass index. Sci Rep 13, 322, doi:10.1038/s41598-022-26572-6 (2023). 36609580 PMC9822919

[35] Peng S. Expression quantitative trait loci (eQTLs) in human placentas suggest developmental origins of complex diseases. Hum Mol Genet 26, 3432–3441, doi:10.1093/hmg/ddx265 (2017). 28854703 PMC5886245

[36] Barbeira A. N. Exploring the phenotypic consequences of tissue Specific gene expression variation inferred from GWAS summary statistics. Nat Commun 9, 1825, doi:10.1038/s41467-018-03621-1 (2018). 29739930 PMC5940825

[37] Watanabe K., Taskesen E., van Bochoven A. & Posthuma D. Functional mapping and annotation of genetic associations with FUMA. Nat Commun 8, 1826, doi:10.1038/s41467-017-01261-5 (2017). 29184056 PMC5705698

[38] Watanabe K., Umicevic Mirkov M., de Leeuw C. A., van den Heuvel M. P. & Posthuma D. Genetic mapping of cell type Specificity for complex traits. Nat Commun 10, 3222, doi:10.1038/s41467-019-11181-1 (2019). 31324783 PMC6642112

